# Neoprzewaquinone A Inhibits Breast Cancer Cell Migration and Promotes Smooth Muscle Relaxation by Targeting PIM1 to Block ROCK2/STAT3 Pathway

**DOI:** 10.3390/ijms24065464

**Published:** 2023-03-13

**Authors:** Guiying Zhao, Yali Ren, Jie Yan, Tingrui Zhang, Peng Lu, Jieting Lei, Huanan Rao, Xin Kang, Zhixing Cao, Fu Peng, Cheng Peng, Chaolong Rao, Yuzhi Li

**Affiliations:** 1 State Key Laboratory of Southwestern Chinese Medicine Resources, School of Pharmacy, Chengdu University of Traditional Chinese Medicine, Chengdu 611137, China; 2 West China School of Pharmacy, Sichuan University, Chengdu 611137, China; 3 R&D Center for Efficiency, Safety and Application in Chinese Materia Medica with Medical and Edible Values, Chengdu University of Traditional Chinese Medicine, Chengdu 611137, China

**Keywords:** Neoprzewaquinone A, PIM1, TNBC, cell migration, ROCK2/STAT3 signaling pathway, glaucoma

## Abstract

*Salvia miltiorrhiza* Bunge (Danshen) has been widely used to treat cancer and cardiovascular diseases in Chinese traditional medicine. Here, we found that Neoprzewaquinone A (NEO), an active component of *S. miltiorrhiza,* selectively inhibits PIM1. We showed that NEO potently inhibits PIM1 kinase at nanomolar concentrations and significantly suppresses the growth, migration, and Epithelial-Mesenchymal Transition (EMT) in the triple-negative breast cancer cell line, MDA-MB-231 in vitro. Molecular docking simulations revealed that NEO enters the PIM1 pocket, thereby triggering multiple interaction effects. Western blot analysis revealed that both NEO and SGI-1776 (a specific PIM1 inhibitor), inhibited ROCK2/STAT3 signaling in MDA-MB-231 cells, indicating that PIM1 kinase modulates cell migration and EMT via ROCK2 signaling. Recent studies indicated that ROCK2 plays a key role in smooth muscle contraction, and that ROCK2 inhibitors effectively control the symptoms of high intraocular pressure (IOP) in glaucoma patients. Here, we showed that NEO and SGI-1776 significantly reduce IOP in normal rabbits and relax pre-restrained thoracic aortic rings in rats. Taken together, our findings indicated that NEO inhibits TNBC cell migration and relaxes smooth muscles mainly by targeting PIM1 and inhibiting ROCK2/STAT3 signaling, and that PIM1 may be an effective target for IOP and other circulatory diseases.

## 1. Introduction

Cell migration is a fundamental physiological function in multicellular organisms [1]. The long distance movement of single cells and cell clusters is essential for embryonic development, wound healing, and immune responses [2]. However, abnormal cell migration is a feature of devastating diseases such as cancer. Cancer cells can migrate in various ways depending on cell morphology, adhesion characteristics and differentiation degree, which performs an essential role in promoting cancer angiogenesis, local invasion and remote diffusion [3]. Despite recent advances in cancer management through cancer screening, chemotherapy, surgery, and radiotherapy, cancer cell migration has proven hard to target therapeutically [4]. Therefore, it is urgent to find an effective drug to target cancer cell metastasis.

Rho-kinase (ROCK), a small guanosine triphosphatase (GTPases), occurs in two isoforms, ROCK1 and ROCK2, and is a downstream effector of Rho A, B, and C. ROCK modulates various cellular processes, including cell contraction, motility, morphology, and proliferation by regulating the actin cytoskeleton [5]. Accumulating evidence suggests that ROCK1/2 upregulation contributes to the malignancy of breast, pancreatic, colorectal, and liver cancer through various signaling factors, including STAT3 and myosin light chain phosphatase 1 (MYPT1), thereby promoting cancer invasion and metastasis [6]. Recently, ROCK2 has become an effective therapeutic target for a variety of disorders, including ophthalmic, cardiovascular, autoimmune, and neurological diseases. Because it is a key regulator of contractile actin fiber and cytoskeletal dynamics, ROCK2 inhibits actin polymerization by generating contractility to mediate smooth muscle movement [7]. Consequently, ROCK2 inhibitors have potential therapeutic value for glaucoma, as well as other diseases such as erectile dysfunction, cardiovascular disease, central nervous system disease, fibrotic disease, inflammation, and diabetes [8,9,10,11,12]. These results prompted us to focus on the role of ROCK2 in cell motility and metastasis.

Glaucoma (prevalence: 3.5%) is a chronic eye disease that causes serious ocular complications that may result in blindness [13]. Glaucoma is primarily associated with pathologically high intraocular pressure (IOP), which is brought on by the retention and buildup of aqueous humor (AH) released by the ciliary body of the eye and dynamically controlled by the trabecular meshwork. The trabecular meshwork is mainly composed of contractile smooth muscle fibers, and therefore has smooth muscle properties. In addition, ROCK2 is abundantly expressed in the trabecular meshwork, making it an ideal target for glaucoma therapy [14]. Small molecule ROCK2 inhibitors, such as Netarsudil and Ripasudil, are used in therapeutic settings to reduce IOP and treat glaucoma by reducing the resistance to AH outflow by relaxing the trabecular meshwork smooth muscle [15,16]. However, since the use of ROCK2 inhibitors to treat glaucoma has adverse side effects, such as conjunctival congestion and subconjunctival congestion [17], safer drugs are urgently needed.

PIM protooncogenes are a family of serine/threonine protein kinases, including PIM1, PIM2, and PIM3, which were first identified in mouse leukemia virus (MuLV)-induced lymphomas [18]. Previous studies have found PIM1 overexpression in solid cancers, particularly in colorectal cancer [19], prostate cancer [20], breast cancer [21], glioblastoma [22], and lung cancer [23] by directly regulating the activities of various proteins involved in cell proliferation, cell cycle arrest, apoptosis, cell autophagy, migration, invasion, and drug resistance [24]. In addition, PIM1 overexpression activates the expression of transcription factors to promote epithelial-mesenchymal transition (EMT) in order to regulate cancer progression and metastasis [25]. Recent studies indicate that targeting PIM1 significantly suppresses breast cancer metastasis as well as the PIM1/STAT3 axis [26]. Since PIM1 and ROCK2 both regulate cell movement, their underlying mechanisms in cell signaling and in smooth muscle movement warrant investigation.

*Salvia miltiorrhiza* Bunge, a member of the Lamiaceae family, was first recorded in the oldest medicine Chinese monograph *Shenlong Bencao Jing* (200–300 AD, Han Dynasty). Its dried root (Danshen) is a *Huoxue Huayu* classic herb that is used for promoting blood circulation by regulating blood vessels and removing lumps, and has long been widely used in the treatment of cardiovascular and cerebrovascular diseases and tumors [27,28]. *S. miltiorrhiza* extracts have been considered as a promising source for a variety of cancers due to their low toxicity and low side effects for decades [29]. Among them, phenanthrenequinone derivatives of *S. miltiorrhiza,* such as Tanshinone IIA and Miltirone, have been reported to inhibit the growth of many types of cancer. In addition to inhibiting cell migration and invasion, Tanshinone IIA and Miltirone can block the cell cycle and induce apoptosis and autophagic death [30,31]. However, phenanthrenequinone derivatives like Neoprzewaquinone A (NEO) have been studied less and deserve further investigation. Therefore, NEO was investigated in this study with the intention of evaluating the effects on MDA-MB-231 cells and smooth muscles. We found that NEO could inhibit the proliferation and migration of TNBC cells by targeting PIM1 and also relieve intraocular pressure by relaxing smooth muscles. Our data revealed a relationship between PIM1 and ROCK2 during cell migration and the modulation of smooth muscle movement.

## 2. Results

### 2.1. NEO Inhibits the Viability and Colony Formation Capacity of MDA-MB-231 Cells

To investigate the effect of NEO on the inhibition of cell growth, we used the MTT assay to determine the effect of NEO on the viability of breast cancer cells (MDA-MB-231 and MCF-7), lung cancer cells (H460 and A549), gastric cancer cells (AGS), liver cancer cells (HEPG-2), ovarian cancer cells (ES-2), myeloma cells (NCI-H929), neuroma cells (SH-SY5Y) and normal breast epithelial cells (MCF-10A). Furthermore, SGI-1776 was set as the positive control. These analyses revealed that NEO suppressed the viability of various cell lines (Table 1), and that this effect was most pronounced in MDA-MB-231 (IC_50_ = 4.69 ± 0.38 μM). Our data showed that SGI-1776, a well-characterized selective PIM1 inhibitor, has good inhibitory activity and selectivity on MDA-MB-231 cells. Thus, MDA-MB-231 cells were selected to further study the underlying mechanisms involved.

**Figure 1:**
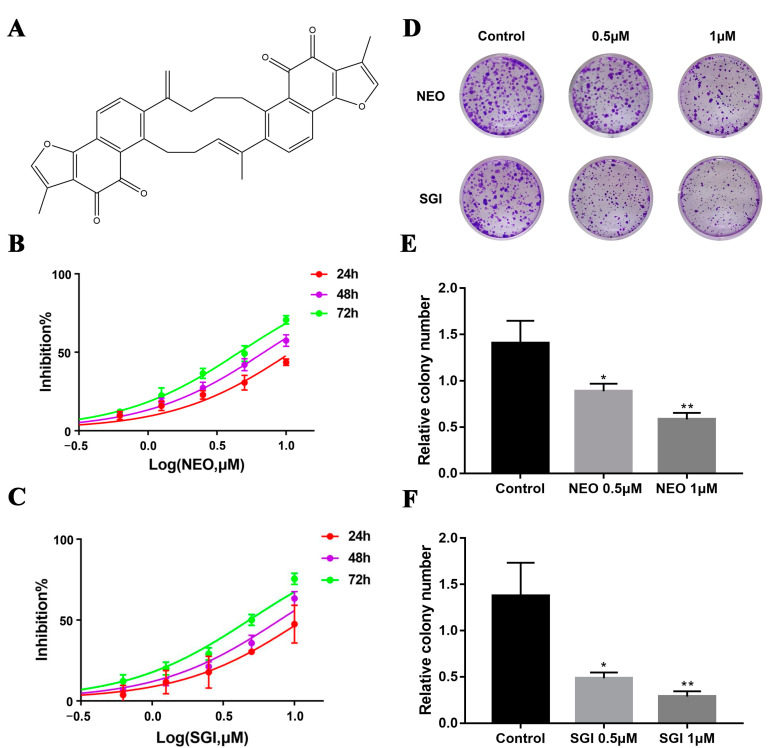
The structure of NEO and inhibitory effect of NEO on the proliferation of MDA-MB-231 cells. (**A**) Molecular structure of NEO. (**B**,**C**) Effects of NEO and SGI-1776 on the proliferation of MDA-MB-231 cells. (**D**) Representative images of MDA-MB-231 cell colonies treated with different concentrations (0, 0.5 and 1 μM) of NEO and SGI-1776. (**E**,**F**) The growth of cancer cell-derived colonies from MDA-MB-231 cells was assessed by determining the number of colonies after NEO and SGI-1776 (0, 0.5 and 1 μM) treatments for 7 days. Each value is the mean ± SD for *n* = 3. * *p* < 0.05, ** *p* < 0.01 versus the control group.

The structure of NEO, a phenanthrenequinone derivative, is shown on Figure 1A. Afterwards, we set NEO to a series of successive concentrations (0.3, 0.6, 1.2, 2.5, 5 and 10 μM) and treated MDA-MB-231 cells for 24 h, 48 h, and 72 h, as well as SGI-1776 (positive control) and assessed their viability using MTT. Furthermore, the time- and concentration-dependent viability curves of NEO and SGI-1776 were drawn, and we calculated the IC_50_ of NEO at 24, 48 and 72 h to be 11.14 ± 0.36, 7.11 ± 1.21, and 4.69 ± 0.38 μM, respectively, whereas that of SGI-1776 was 11.74 ± 0.45, 8.03 ± 0.41, and 4.90 ± 0.21 μM, respectively (Figure 1B,C), indicating that NEO potently inhibited the cell proliferation of MDA-MB-231. In addition, we investigated the effect of NEO and SGI-1776 on the colony formation ability of MDA-MB-231 cells, which indicated that both NEO and SGI-1776 effectively suppress colony formation by MDA-MB-231 at low concentrations compared with the negative control group (Figure 1D–F). Together, these data showed that NEO and SGI-1776 could retard MDA-MB-231 cell growth in vitro.

### 2.2. NEO Inhibits Migration and Invasion of MDA-MB-231 Cells

**Figure 2:**
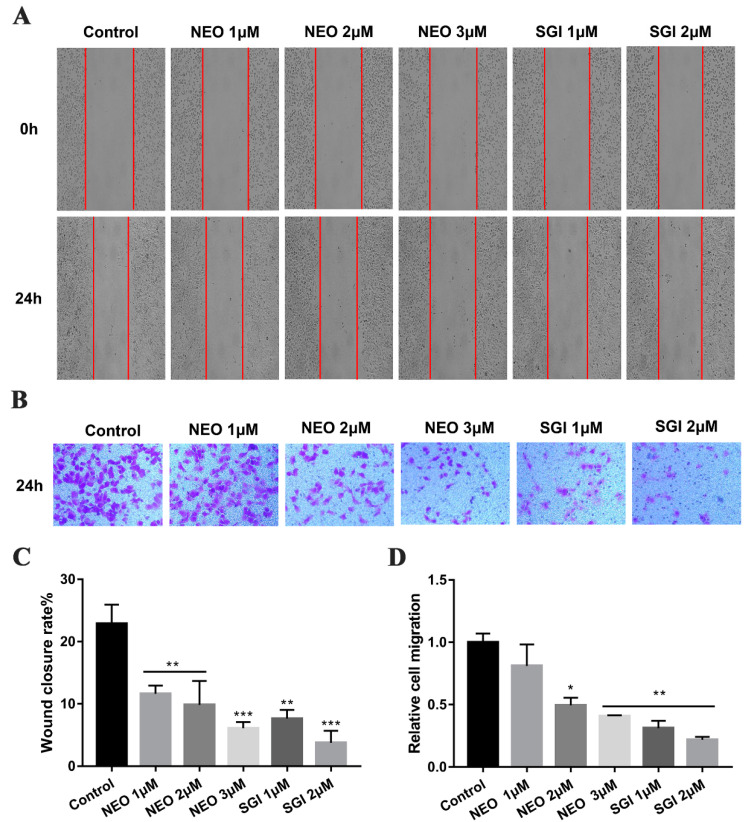
NEO and SGI-1776 inhibits cell migration and invasion in MDA-MB-231 cells. (**A**) The migration of MDA-MB-231 cells was assessed by the wound healing assay. Representative images of wound closure were taken at 0 h and 24 h (magnification, ×40). (**B**) Invasions of MDA-MB-231 cells were assessed by a Transwell assay. Representative images of invasive cells were obtained at 24 h (magnification, ×200). (**C**) The wound healing percentage of MDA-MB-231 cells was calculated. (**D**) Relative number of invasive cells. Each value is the mean ± SD for *n* = 3. * *p* < 0.05, ** *p* < 0.01, *** *p* < 0.001 versus control group.

As we know that MDA-MB-231 cells exhibit high migration and invasion capacity, and we have previously demonstrated that NEO can suppress the cell survival and colony formation, therefore we investigated whether NEO was involved in reducing MDA-MB-231 cells metastasis. The wound healing analysis was used to assess the subcytotoxic concentration of NEO (1, 2 and 3 μM) to anti-migration. This analysis revealed that relative to the negative control, treatment with NEO and SGI-1776 for 24 h significantly suppressed migration by MDA-MB-231 cells (Figure 2A,C). Furthermore, in agreement with these results, similar data were obtained upon Transwell analysis of the effect of NEO on invasion, which showed that treatment with NEO and SGI-1776 for 24 h significantly suppressed invasion by MDA-MB-231 (Figure 2B,D). These data indicate that NEO could inhibit the migration and invasion of MDA-MB-231 cells.

### 2.3. NEO Inhibits PIM1 at the Kinase Level and Interacted with PIM1 via a Molecular Docking Simulation

**Figure 3:**
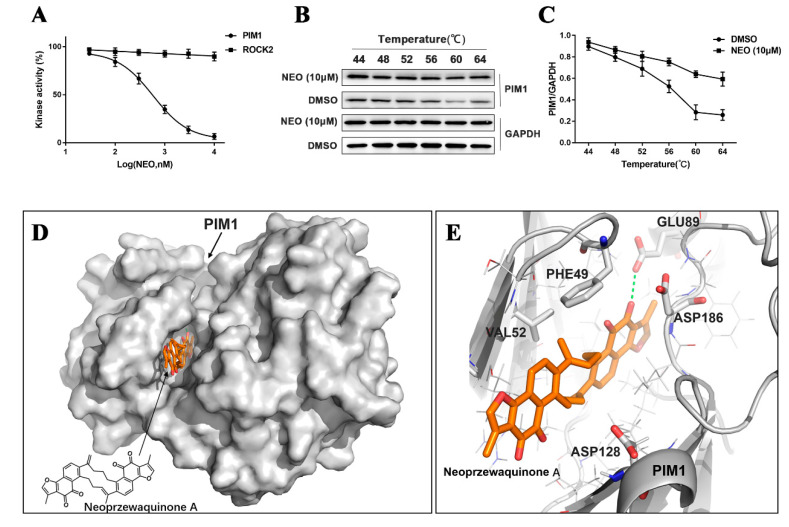
PIM1 is the potential target of NEO. (**A**) Inhibitory effects of NEO on PIM1 and ROCK2 kinase. (**B**,**C**) CETSA-WB assay to confirm that NEO targeted PIM1 proteins. (**D**) Three-dimensional structure of the PIM1 receptor and NEO ligand. (**E**) The protein backbone of PIM1 receptor is presented as a cartoon. Each value is the mean ± SD for *n* = 3.

In order to study the inhibitory mechanism of NEO, we performed an in vitro kinase assay to confirm the effect of NEO. The ADP-Glo^TM^ Kinase Assay was used to monitor the activities of PIM1 kinase. Our results showed that NEO significantly inhibited PIM1 activities. The inhibitory effects increased with increasing NEO concentrations. The IC_50_ of NEO was 0.56 μM, while there was almost no inhibition of ROCK2 (Figure 3A). Thus, NEO inhibited PIM1 activities at the enzyme level, consistent with the simulation results of molecular docking.

In addition, we performed the CETSA assay to identify the binding of NEO to PIM1 by detecting the increased stability of target proteins to heat-induced precipitation by western blot. Compared to DMSO blank, NEO (10 μM) enhanced the thermal stability of PIM1 by about 4 °C. It was established that NEO incubation stabilized PIM1 in heat-denatured MDA-MB-231 cells (Figure 3B,C).

Additionally, we utilized a computational docking model to validate the potential interaction between NAO and PIM1. In Figure 3D, the surface of the PIM1 receptor and the cartoon defining the protein backbone are grey. The structure of the NEO ligand was shown in stick representation (orange). In Figure 3E, the five key residues (VAL52, PHE49, GLU89, ASP186, ASP128) in the binding cavity of the PIM1 receptor are colored in grey, while the carboxyl of the GLU89 residue in the active site shows hydrogen bond interactions with the NEO ligand. The hydrogen bond was shown by the green dashed line. A molecular docking simulation analysis showed that compound NEO can bind the PIM1 receptor, and the main binding force is based on hydrogen bond interactions between the GLU89 residue of the PIM1 receptor with NEO, implying that NEO has considerable inhibitory effects on PIM1. These results indicate that NEO can directly inhibit PIM1 kinase activity.

### 2.4. NEO Inhibits the PIM1/ROCK2/STAT3 Signaling Pathway and EMT Progression in MDA-MB-231 Cells

**Figure 4:**
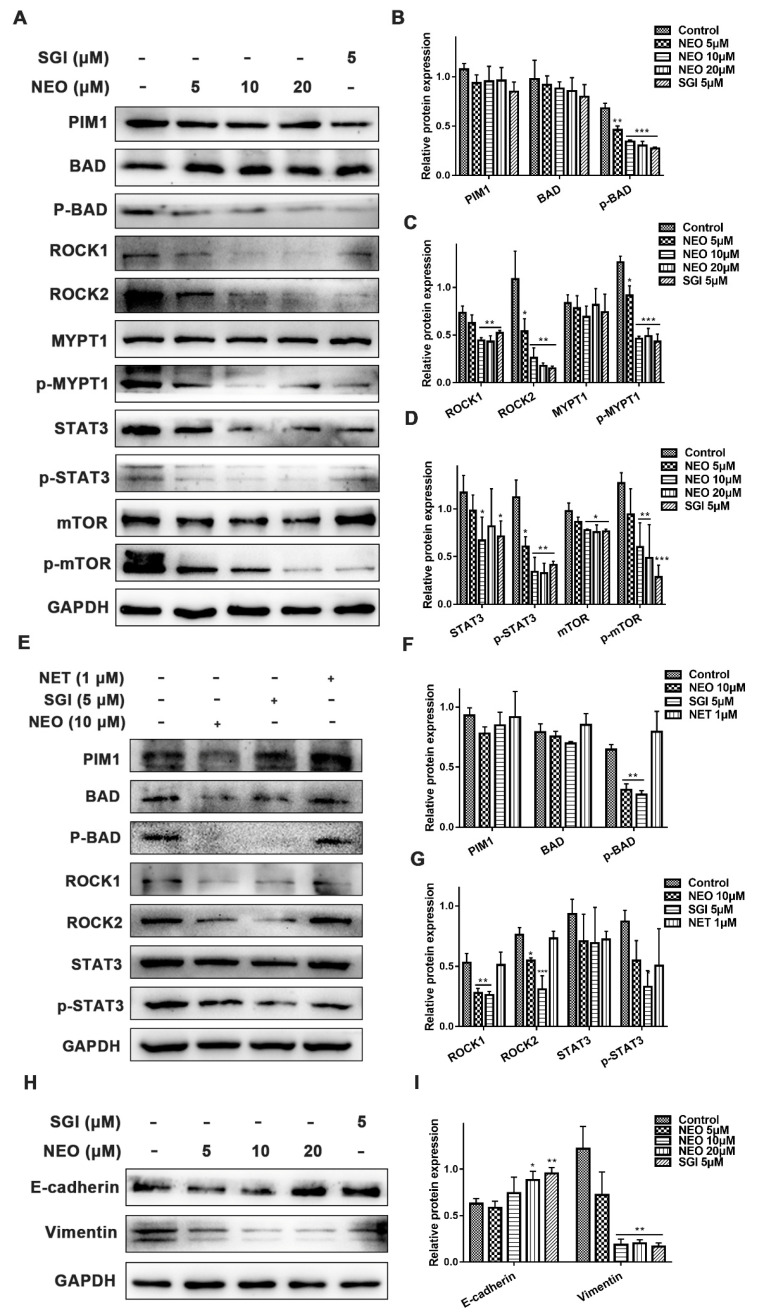
NEO and SGI-1776 inhibited the PIM1/ROCK2/STAT3 signaling pathway and EMT progression. (**A**) Representative western blot image for PIM1 signaling in MDA-MB-231 cells after exposure to NEO and SGI-1776 for 20 h. (**B**–**D**) Optical density analysis chart of protein bands. The data shows that NEO and SGI-1776 significantly inhibited PIM1, ROCK1, ROCK2 and p-STAT3 proteins, and down-regulated the expressions of downstream signaling factors (p-BAD, p-MYPT1 and p-mTOR). (**E**) Validation of the pathway relationship between PIM1 and ROCK2. (**F**,**G**) Optical density analysis chart of protein bands. The data shows that PIM1 is upstream of ROCK2. (**H**,**I**) Western blot image of E-cadherin and Vimentin protein expressions in MDA-MB-231 cells after exposure to NEO and SGI-1776 for 20 h. Each value is the mean ± SD for *n* = 3. * *p* < 0.05, ** *p* < 0.01, *** *p* < 0.001 versus control group.

To clarify the underlying mechanisms of the inhibitory effects of NEO in MDA-MB-231 cells migration, we determined the expressions of PIM1 and ROCK2 signaling pathway-related proteins by western blot analysis. MDA-MB-231 cells were treated with NEO and SGI-1776 for 20 h and cell lysates were prepared. We observed that the expression of ROCK1, ROCK2, p-MYPT1, p-mTOR and p-STAT3, proteins belonging to the PIM1 signaling pathways, were suppressed in a dose-dependent manner. However, total protein levels of PIM1, BAD, MYPT1, mTOR and STAT3 did not significantly change (Figure 4A–D). Later, we used the PIM1 inhibitor (SGI-1776) and the ROCK2 inhibitor (Netarsudil) to verify the relationship between PIM1 and ROCK2 on signaling pathways. The inhibition of PIM1 suppressed ROCK2 expressions, indicating that ROCK2 is a downstream protein of PIM1, and ROCK2 expressions are regulated by PIM1 (Figure 4E–G). These findings indicated that NEO inhibits the PIM1/ROCK2/STAT3 signaling pathway in MDA-MB-231 cells.

EMT plays a key role in cancer progression, invasion and metastasis, and is a way by which cancer cells gain aggressive properties [23]. To elucidate as to whether NEO has a role in the EMT process during MDA-MB-231 cell migration, a western blot assay was applied to investigate the expression of the EMT markers E-cadherin and vimentin. The results showed that compared with the control, treatment with NEO can dose-dependently up-regulated the activity of E-cadherin and down-regulated the activity of Vimentin. The same results were found in the SGI-1776 treatment group (Figure 4H,I). These findings showed that NEO dose-dependently inhibits the EMT progression in MDA-MB-231 cells.

### 2.5. NEO Induces G0/G1 Phase Arrest and Apoptosis in MDA-MB-231 Cells

**Figure 5:**
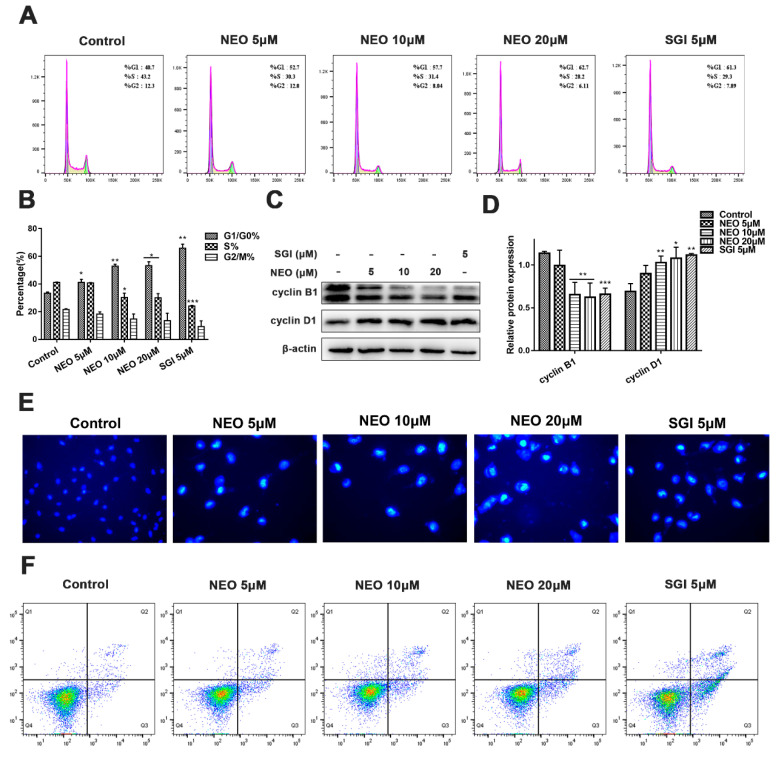
NEO and SGI-1776 induced cell cycle arrest and apoptosis of MDA-MB-231 cells. (**A**,**B**) Cell cycle distribution map of MDA-MB-231 cells treated with NEO and SGI-1776 for 24 h was detected by flow cytometry. NEO (concentration-dependent manner) and SGI-1776 significantly increased the ratio of cells in the G1/G0 phase. (**C**,**D**) Western blot image. Treatment of MDA-MB-231 cells with NEO for 24 h significantly down-regulated the expressions of cyclin B1 and up-regulated the expressions of cyclin D1. (**E**) MDA-MB-231 cells were treated with NEO and SGI-1776 for 24 h, stained with Hoechst 33258, and imaged. Cell apoptosis can be observed (magnification, ×200). (**F**) Apoptosis was detected by annexin V-FITC/PI staining. Treatment of MDA-MB-231 cells with NEO and SGI-1776 for 24 h induced apoptosis. Each value is the mean ± SD for *n* = 3. * *p* < 0.05, ** *p* < 0.01, *** *p* < 0.001 versus control group.

To further confirm the anticancer potential of NEO, we used flow cytometry to examine whether the inhibition of PIM1 expression could contribute to cell cycle arrest and apoptosis. After 24 h of exposure to NEO and SGI-1776, MDA-MB-231 cells were arrested in the G0/G1 phase in a concentration-dependent manner compared to the control group, and the percentage of cell arrest was significantly diminished in the S phase. In particular, treatment with 20 μM NEO significantly increased the proportion of cells in the G0/G1 phase from 37.35 ± 4.74% to 59.00 ± 5.23%, while the abundance of cells in the S phase decreased from 42.20 ± 1.41% to 30.20 ± 2.83%. Similarly, the SGI-1776 treatment group significantly induced cell cycle arrest in the same phase (Figure 5A,B). In order to understand the mechanism that regulates the cell cycle upon treatment with NEO, a Western blot assay was carried out to analyze the expressions of the key proteins cyclin B1 and cyclin D1, which are associated with the cell cycle. The results indicated that the cyclin B1 expression level was apparently lower than that in the control group, while cyclin D1 was up-regulated in a dose-dependent manner (Figure 5C,D), the finding of which demonstrated that NEO induces cell cycle arrest at the G0/G1 phase via downregulating the expression of cyclin B1.

After being exposed to NEO and SGI-1776 for 24 h, the Hoechst 33,258 staining method was used to assess typical nuclear morphological features. The pictures showed that upon increasing the NEO concentrations, the morphology of the nucleus begins to shrink, and the number of cells with bright blue fluorescence increased (Figure 5E). Furthermore, flow cytometry was used to assess apoptosis. The results revealed that upon an increasing NEO concentration, the number of apoptotic cells gradually increased. In particular, treatment with 20 μM NEO and 5 μM SGI-1776 induced apoptosis, and the cell proportions increased from 5.18 ± 1.64% to 19.62 ± 1.78% and 25.88 ± 0.67%, respectively (Figure 5F). These data showed that after NEO treatment, MDA-MB-231 cells exhibited a higher level of apoptosis.

### 2.6. NEO Induces Autophagy in MDA-MB-231 Cells

**Figure 6:**
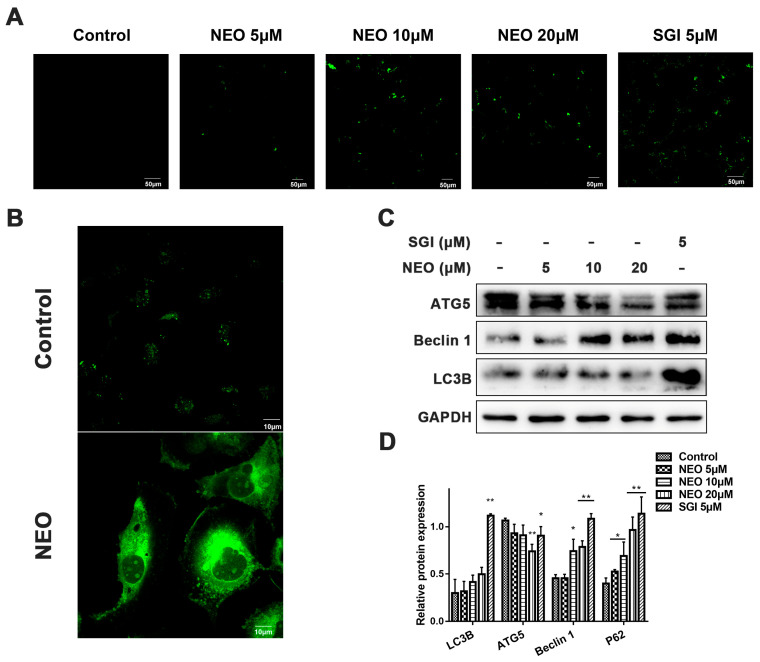
NEO and ibrutinib-induced autophagy. (**A**) Confocal laser-stained image of MDC, showing that MDA-MB-231 cells underwent autophagy after 24 h of exposure to NEO and SGI-1776 (there was an increase in autophagosomes (green fluorescence)) (scale bar, 50 μm). (**B**) Partial enlarged images of the control and NEO groups stained with MDC. The NEO-induced autophagosomes can be clearly seen (scale bar, 10 μm). (**C**,**D**) Western blot images. Treatment of MDA-MB-231 cells with NEO and SGI-1776 for 24 h significantly upregulated the expressions of Beclin1 and LC3B, while downregulating the expressions of ATG5. Each value is the mean ± SD for *n* = 3. * *p* < 0.05, ** *p* < 0.01 versus control group.

To further confirm the anticancer potential of NEO, we used MDC staining to examine whether the inhibition of PIM1 expression could contribute to cell autophagy. After 24 h of exposure to NEO and SGI-1776, MDC staining was performed to detect autophagy. As shown in the pictures, MDA-MB-231 cells exhibited green fluorescence in the treatment groups, indicating that the cells underwent autophagy. Furthermore, the enlarged image showed that NEO activated the autophagosomes in MDA-MB-231 cells (Figure 6A,B). LC3B, Beclin1 and ATG5 are key regulators of autophagy and are well-studied targets. In this study, we performed a Western blot analysis to evaluate the effects of NEO on the expression of the of LC3B, Beclin1 and ATG5 proteins. The results disclosed that NEO could induce the autophagy of MDA-MB-231 cells by up-regulating the expressions of Beclin1 and LC3B, while down-regulating the expressions of ATG5 (Figure 6C,D). These findings suggested that the autophagy induced by NEO is related to the inhibition of ATG5 expression.

### 2.7. NEO Suppresses IOP in Normal NZW Rabbits

**Figure 7:**
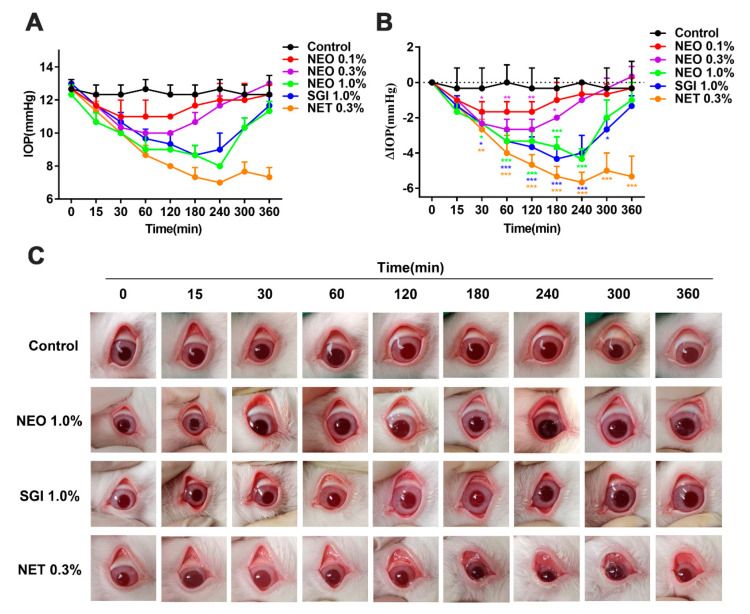
NEO, SGI-1776 and NET decreased IOP. (**A**) IOP levels prior to treatment and at 15, 30, 60, 120, 180, 240 and 300 min after treatment. (**B**) Changes in IOP (ΔIOP) at 15, 30, 60, 120, 180, 240 and 300 min after treatment; (**C**) A representative image of conjunctival hyperemia at 0, 15, 30, 60, 120, 180, 240 and 300 min after dose administration. Each value is the mean ± SD for *n* = 3. * *p* < 0.05, ** *p* < 0.01, *** *p* < 0.001 versus control group.

To assess the IOP-suppressing effects of NEO (0.1%, 0.3%, 1.0%), SGI-1776 (1.0%), and NET (0.3%), IOP measurements were performed at indicated times before and after the administration of each eye drop in ocular normotensive NZW rabbits. Within a certain time, each administration group showed a time-dependent decrease in IOP. All concentrations of NEO reduced IOP levels, and the decrease in intraocular pressure was observed from 15 min after administration and continued for 3–4 h (Figure 7A). The maximum intraocular pressure reduction (ΔIOP) observed at 60 min after the administration of NEO (0.3 %) was 2.67 ± 0.58 mmHg and thereafter increased to reach the baseline. In the SGI-1776 (1.0%) and NET (0.3%) experimental groups, the observed ΔIOP were 4.33 ± 0.58 and 5.67 ± 0.58 mmHg, respectively. In the NEO (1.0%) experimental group, IOP decreased from 12.67 ± 0.58 at baseline to 8.34 ± 0.58 mmHg at 240 min. The ΔIOP was 4.33 ± 0.58 mmHg and increased thereafter. Compared with the untreated group, the IOP of normal NZW rabbits in the NEO (1.0%), SGI-1776 (1.0%) and NET (0.3%) groups significantly decreased, while the effects of NEO (1.0%) and SGI-1776 (1.0%) were weaker than those of NET (0.3%) (Figure 7B).

Furthermore, mild conjunctival hyperemia was observed at 240 min in the NEO (1.0%) group, whereas conjunctival hyperemia in SGI-1776 (1.0%) treated eyes started after 30 min post-dose emerged and persisted to the end; conjunctival hyperemia was severe in the NET (0.3%) treated group (Figure 7C). Thus, NEO induced minimal conjunctival hyperemia compared to NET. As a ROCK2 inhibitor, NET directly inhibited the contraction of trabecular meshwork cells and reduced IOP; therefore, we suspected that NEO may have the same mechanisms for reducing IOP in NZW rabbits.

### 2.8. NEO Relaxes Smooth Muscles by Inhibiting the PIM1/ROCK2/STAT3 Signaling Pathway

**Figure 8:**
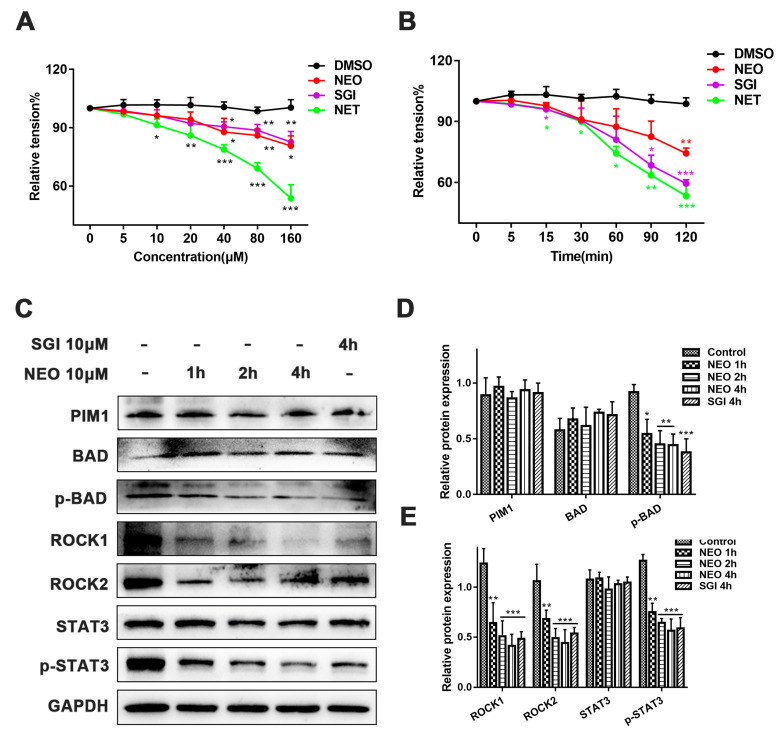
NEO, SGI-1776 and NET decreased IOP and dilated the smooth muscles. (**A**) Vasorelaxant effects of NEO, SGI-1776 and NET (concentration-dependent manner) on rat thoracic aorta rings precontracted with 60 mM KCL. (**B**) Vasorelaxant effects of NEO, SGI-1776 and NET (10 μM; time-dependent manner) on rat thoracic aorta rings precontracted with 60 mM KCL. (**C**) Representative western blot image for PIM1 signaling in smooth muscles after exposure to 10 μM NEO and SGI-1776. (**D**,**E**) Optical density analysis chart of protein bands. The data shows that NEO and SGI-1776 significantly inhibited PIM1, ROCK1, ROCK2, p-BAD and p-STAT3 proteins. Each value is the mean ± SD for *n* = 3. * *p* < 0.05, ** *p* < 0.01, *** *p* < 0.001 versus the control group.

To confirm whether the IOP-lowering effects of NEO are accomplished via relaxation-related smooth muscles, pre-contracted rat isolated thoracic aortic rings were treated with NEO. We found that 10 μM NEO, SGI-1776 and NET dose- and time-dependently significantly relaxed 60 mM KCl precontracted rat thoracic aortic rings (Figure 8A,B). To further determine the mechanism of smooth muscle relaxation by NEO, we quantified the expression levels of proteins related to the PIM1/ROCK2/STAT3 pathway by western blot. The results showed that the expression levels of PIM1, ROCK1, ROCK2, p-BAD and p-STAT3 were significantly reduced after exposure to 10 μM NEO and SGI-1776, and NEO time-dependently inhibited the expression of these signaling molecules. These data suggested that NEO relaxed smooth muscle by inhibiting the PIM1/ROCK2/STAT3 signaling pathway (Figure 8C–E).

## 3. Discussion

Globally, breast cancer accounts for the most common and deadly cancer among women, making it the leading cancer in the world, already affecting 2.26 million women in 2020 [32]. TNBC lacks specific biomarkers (ER-, PR-, HER2-) and is highly aggressive, extensively intra-cancer heterogeneous, and frequently drug-resistant compared to other breast cancer subtypes [33]. Patients with TNBC have the worst prognostic outcomes and are also prone to metastasis and recurrence after treatment, thus, the development of drugs targeting migration is imminent.

Herbal medicines have been used for cancer treatment because of their natural nature and low toxicity [34,35]. *S. miltiorrhiza* has a long history of medicinal use in China, and its active constituents have shown potent efficacies against breast cancer [36]. But as the active ingredient in *S. miltiorrhiza*, NEO has been studied less. According to the MTT assays and colony formation experiments, we discovered that NEO dose-dependently inhibited the survival and proliferation of various cancer cell lines, with higher inhibitory activities against TNBC MDA-MB-231 cells (IC_50_ is 4.69 ± 0.38 μM) compared to other cell lines. In addition, according to the results of wound-healing experiments and a Transwell assay, we discovered that NEO had inhibitory effects on the migration and invasion of MDA- MB-231 cells. The specific pathomechanisms should be investigated.

In the past decades, targeted therapies have entered the forefront in oncology [37]. To elucidate the specific molecular mechanisms by which NEO inhibits the migration of TNBC cells, we explored the targets of NEO. According to the results of kinase assays, we found that after treatment with NEO, PIM1 kinase activities were selectively inhibited (IC_50_ is 0.56 μM). Molecular docking simulations also demonstrated that NEO can inhibit PIM1 kinase activities by flexibly penetrating the PIM1 pocket to form multiple interactions with five residues (VAL52, PHE49, GLU89, ASP186 and ASP128). It has been found that PIM1 kinases is over-expressed in many malignant cancers, where they boost the cell proliferation, and participate in migration and metastasis [38]. ROCK is also involved in multiple steps of cancer progression and has been shown to regulate cell motility, promote migration and invasion, as well as cancer cell metastasis [39]. Both PIM1 and ROCK2 promote cell migration in cancer, and there may be some associations. The kinase assays showed that NEO had no inhibitory activities on ROCK2, indicating that NEO does not act directly on ROCK2. Therefore, we hypothesized that they might be upstream and downstream in the regulation of cell motility. Therefore, we used the PIM1 inhibitor (SGI-1776) and ROCK2 inhibitor (Netarsudil) to investigate the relationship between the two. Western blot results showed that the inhibition of PIM1 suppressed ROCK2 expressions, suggesting that ROCK2 is a downstream protein of PIM1 and that ROCK2 expressions are regulated by PIM1.

STAT3 is central to anti-cancer, and participates in cancer progression, differentiation and metastasis [40]. The ROCK2/STAT3 pathway has become a major research direction in tumor immunity [41]. In our present studies, the phosphorylation levels of BAD at Ser112 decreased with increasing NEO concentrations, while the expressions of downstream proteins (ROCK1/2, p-MYPT1, p-STAT3 and p-mTOR) were significantly reduced. In addition, as a hallmark of cancer progression, EMT enables cancer cells to acquire stem cell-like and anti-cancer phenotypes, allowing them to invade and migrate [42]. We established that NEO inhibited EMT progression by suppressing the expressions of waveform proteins Vimentin and up-regulating E-cadherin expressions, thus preventing cell metastasis. These data suggest that NEO exerts good anti-proliferative, migratory and invasive effects on TNBC cells by targeting PIM1 to block the ROCK2/STAT3 signaling pathway.

Studies have confirmed that PIM1 is involved in the cell cycle, apoptosis, and autophagy processes [43,44]. As the cell cycle progresses, cyclins play an extremely vital role in controlling cell proliferation and differentiation [45]. Cyclin B1 and cyclin D1 are essential regulators of the G1-S cell cycle transition, and in this study, we obviously observed western blot results that indicated that NEO attenuated the expression of cyclin B1 in MDA-MB-231 cells, thus keeping cells from progressing into the S phase, consistent with the flow cytometry results, elevating the proportion of G0/G1 phase cells. Furthermore, the flow cytometry and Hoechst 33,258 staining results revealed that NEO could induce concentration-dependent apoptosis in MDA-MB-231 cells. Our Western blot results with NEO-treated cells showed that the expression of ATG5, LC3B and Beclin1, the key executors of cell autophagy, was decreased or upregulated. These findings suggested that NEO was capable of inducing G0/G1 phase arrest, apoptosis and autophagy in MDA-MB-231 cells and exerting promising anti-tumor effects.

In addition to playing an integral role in cancer development and metastasis, ROCK2 regulates the cellular contraction of smooth muscle cells and non-muscle cells [46]. Studies have shown that ROCK2 inhibitors can lower IOP by modulating actin contractile fibers, thereby treating glaucoma. Given that ROCK2 inhibitors inhibit smooth muscle contraction, as upstream of ROCK2, we hypothesized that PIM1 might also have relaxing effects on smooth muscles. The IOP test revealed that, as with Netarsudil, NEO and SGI-1776 suppressed IOP in normal rabbits and resulted in mild conjunctival congestion. We then used precontracted isolated thoracic aortic rings to verify the specific mechanisms by which NEO inhibits IOP. It was found that NEO relaxes vascular smooth muscles and exhibits concentration-dependent and time-dependent effects, implying that NEO inhibits IOP by targeting the diastole of trabecular meshwork smooth muscles.

There have been various advances in the treatment of chronic graft-versus-host disease (cGVHD) in recent years. As an immune-mediated inflammatory and fibrotic disease, cGVHD causes tissue damage and multisystem organ involvement, severely affecting the quality of life of patients. With the introduction of targeted therapies, cGVHD management is still developing. ROCK2 is a key regulator of immunomodulation and fibrosis, and controls protofibrosis by regulating Th17/regulatory T cell balance as well as pathways, becoming a therapeutic target for cGVHD [47]. In a IIa clinical trial for the treatment of cGVHD, the oral selective ROCK2 inhibitor (Belumosudil) improved ORR as well as overall survival outcomes, and showed an improved quality of life [48]. The PIM1 kinase is highly expressed in idiopathic fibrotic lungs and enhances fibrogenic activation [49]. The suppression of PIM1 inhibits the survival, differentiation and proliferation of B and T cells and suppresses the activation of NK cells to downregulate immune responses. Therefore, the PIM1/ROCK2 signaling pathway might be regulated in pathological processes of immunity, as well as fibrosis, and has the potential for the treatment of related diseases. Given that active components in *S. miltiorrhiza* have anti-inflammatory and other pharmacological activities, we hypothesized that NEO-like components in *S. miltiorrhiza* may be the key components in *S. miltiorrhiza* that exert immunomodulation and blood circulation functions, which deserve attention in herbal quality research.

## 4. Materials and Methods

### 4.1. Drugs and Materials

Neoprzewaquinone A (purity ≥98%) was obtained from Herbpurify (Chengdu, China). SGI-1776 and Netarsudil (purity ≥98%) were purchased from Selleck (Chengdu, China). RPMI 1640, DMEM, DMEM/F12 media and Fetal Bovine Serum (FBS) were acquired from Gibco (NewYork, NY, USA). Paraformaldehyde (PFA; 4%) was purchased from Biosharp (Hefei, China). The BCA protein assay kit, cell cycle analysis kit, annexin V-FITC/PI apoptosis detection kit and Hoechst 33258 were obtained from Beyotime (Shanghai, China). The trypsin, crystal violet (1%), and Mondansylcadaverine (MDC) assay kit were purchased from Solarbio (Beijing, China). The primary antibodies against ROCK1, ROCK2, mTOR/p-mTOR, MYPT1/p-MYPT1, PIM1, BAD/p-BAD, STAT3/p-STAT3, E-cadherin, Vimentin, cyclin B1, cyclin D1, Beclin1, ATG5, LC3B, GAPDH, *β*-actin, and HRP-conjugated secondary antibodies were purchased from Affinity (Jiangsu, China). The electrochemiluminescence (ECL) western blot detection kit was purchased from Abbkine (Redlands, CA, USA).

### 4.2. Cell Culture

Human cell lines MDA-MB-231, HEPG-2, NCI-1299, AGS, MCF-7, H460, ES-2, A549, MCF-10A and SH-SY5Y cells were obtained from the ATCC (Manassas, VA, USA). Cells were cultured in the presence of 5% CO_2_ and 37 °C in RPMI 1640, DMEM, and DMEM/F12 medium supplemented with 1% (*v/v*) penicillin-streptomycin and 10% (*v/v*) FBS.

### 4.3. Cell Viability Assays

Cell viability was evaluated using the MTT assay. Specifically, 3 × 10^3^ cells (MDA-MB-231, HEPG-2, NCI-1299, AGS, MCF-7, H460, ES-2, A549, MCF-10A and SH-SY5Y; in 100 μL of media) were seeded into 96-well plates and cultured until cell apposition. After treating the cells with different concentrations of NEO and SGI-1776 (0.3, 0.6, 1.2, 2.5, 5, and 10 μM) for 24 h, 48 h, and 72 h, respectively, the control group was set. Subsequently, the cell viability was measured by the MTT assay according to the manufacturer’s instructions.

### 4.4. Colony Formation Assay

The generation of single cell suspensions was performed by trypsinization. Next, 100 MDA-MB-231 cells were seeded into 24-well plates and cultured for 7 days at complete media with different concentrations of NEO and SGI-1776. After fixing with 4% PFA for 20 min, the colonies were then stained for 15 min at room temperature with 1% crystal violet. Following that, the colonies were counted in three random wells.

### 4.5. Wound Healing Assay

To create a “wound” in the cell monolayer, 5 × 10^5^ MDA-MB-231 cells were cultured to 100% confluence. Next, we held a sterile 200 μL pipette absolutely vertical to the plate’s surface, and then a wound was created by scratching the monolayer. The cells were rinsed three times with PBS to remove non-adherent cells, and they were cultured and imaged at 0 and 24 h after wounding. The wound closure rate was analyzed using the formula: healing area/initial scratch area ×100%.

### 4.6. Transwell Assay

Single cell suspensions were generated by trypsinization in serum-free medium. 1 × 10^5^ MDA-MB-231 cells were seeded into the upper Matrigel-coated chamber, and the lower one was filled with complete media (with 10% FBS). After being treated with NEO (1, 2 and 3 μM) or SGI-1776 (1 and 2 μM), The cells were then cultured for 24 h before being removed from the upper chamber. We used 4% PFA to fix the cells that had infiltrated the lower chamber, and they were stained for 10 min with 1% crystal violet, and counted in three randomly selected fields of view. The mean number of invading cells per field of view was regarded as the cell’s invasion ability.

### 4.7. Kinase Inhibition Assay

The ADP-Glo^TM^ kinase assay was performed to evaluate the ability of NEO to inhibit PIM1 kinase activity. To achieve a final concentration of 10M, pure ATP was serially diluted. NEO was dissolved in DMSO and used at 0.3, 1, 3, 10 and 30 μM final concentrations. As a negative control, DMSO (vehicle) was used. Following that, a 5 μL kinase reaction was carried out using 1×kinase buffer as directed by the manufacturer. After 60 min at room temperature, 5 μL of ADP-Glo reagent was added, and the reaction was incubated for another 40 min. Finally, 10 μL of kinase assay reagent was added, and the reaction was incubated at room temperature for 30 min before the kinase activity was determined using a Luminoskan Ascent plate reader.

### 4.8. Cellular Termal Shift Assay

We performed the cellular thermal shift assay (CETSA) to determine that NEO was targeting PIM1. After treating with DMSO or NEO (10 μM) for 2 h, MDA-MB-231 cells were then lysed with an RIPA lysis solution containing a cocktail of protease and phosphatase inhibitors. Later, the protein solution was divided into six equal parts. The lysates were then heated for 3 min each at 44, 48, 52, 56, 60, and 64 °C, and then incubated at 4 °C for 3 min. The soluble supernatant was incubated for 5 min at 100 °C with 1× loading buffer before being stored at −80 °C for western blot.

### 4.9. Molecular Docking

All molecular docking simulations were performed using the GOLD program in Discovery Studio (Accelrys, San Diego, CA, USA). GOLD is a genetic algorithm-based molecular docking method that uses the CHARMm force field. All molecules were prepared before docking using the prepare ligands protocol in Discovery Studio. PIM1′s X-ray diffraction crystal structure used for molecular docking analysis was from the Protein Data Bank (PDB ID: 1XWS; www.rcsb.org, accessed on 28 September 2022). Finally, molecular docking simulation figures were constructed using pymol (http://pymol.org/educational v2.x, accessed on 28 September 2022).

### 4.10. Western Blot Analysis

The total protein was obtained by lysing cells in a chilled RIPA buffer. The BCA protein assay kit was then used to quantified protein concentration. A western blot analysis was performed as previously described [50]. The primary antibodies against PIM1, BAD/p-BAD, ROCK1, ROCK2, MYPT1/p-MYPT1, STAT3/p-STAT3, mTOR/p-mTOR, E-cadherin, Vimentin, cyclin B1, cyclin D1, Beclin1, ATG5, LC3B, GAPDH, and β-actin were detected.

### 4.11. Apoptosis Analysis

MDA-MB-231 cells were treated for 24 h with NEO at 5, 10 and 20 μM, and SGI-1776 at 5 μM to detect apoptosis. Then, cells were collected and incubated in the dark at 37 °C with 500 μL of 10μg/mL Hoechst 33258 staining solution for 10 min. Following that, the cells were examined under a fluorescent microscope (Leica DMI3000B, Germany).

The apoptosis rate was determined using an Annexin V-FITC apoptosis detection kit. MDA-MB-231 cells were treated and harvested as described above for Hoechst 33258 staining. Following that, binding buffer, Annexin V-FITC and PI were added to the suspension according to the instructions, and they were then mixed and incubated in the dark for 15 min. The apoptosis rate was assessed and analyzed by flow cytometry (Becton Dickinson, NJ, USA).

### 4.12. Cell-Cycle Analysis

For cell cycle analysis, flow cytometry tests were used. MDA-MB-231 cells were treated with NEO at 5, 10 and 20 μM and SGI-1776 at 5 μM for 24 h, and harvested as described above. They were then incubated overnight at 4 °C with 70% ethanol. Next, the cells were suspended in 500 μL of binding buffer, 10 μL of RNase A and 25 μL of PI, and incubated for 30 min in the dark. After that, the cell cycle stage was assessed immediately by using flow cytometry.

### 4.13. Autophagy Analysis

MDC is an eosinophilic stain that is commonly used as a special marker stain to detect autophagosome formation. To examine autophagy, MDA-MB-231 cells were seeded into confocal dishes before treating them with NEO at 5, 10 and 20 μM and SGI-1776 at 5 μM for 24 h. Next, the cells were incubated with an MDC staining solution at room temperature for 1 h. The cells were then washed with PBS three times and examined by a confocal fluorescence microscope (Olympus FV1200, Japan).

### 4.14. Intraocular Pressure Assay

Male NZW rabbits (body weight 2–2.5 kg) and male rats (body weight 180–220 g) were obtained from the animal experiment center of Chengdu University of Traditional Chinese Medicine. Animals were housed in separate cages under controlled temperature (22–23 °C) and a 12 h light/dark cycle with ad libitum access to food and water. All animal experiments adhered to the guidelines of the animal Ethics Committee of Chengdu University of Traditional Chinese Medicine (ethics code: 2022-51).

The rabbits were randomly divided into a control group (received sterile water), the NEO group (received 0.1%, 0.3%, or 1.0% NEO), the SGI-1776 group (received 1.0% SGI-1776), and the Netarsudil (NET) group (received 0.3% NET). NEO, SGI-1776, and NET were dissolved in sterile water containing 3% polypropylene glycol 400 (PEG-400), and 50 µL of each suspension was topically administered to one eye of each rabbit. IOP measurements (three replicate measurements per eye) were taken using a handheld rebound tonometer (FA800 Vet, Fuan, Shanghai, China) at 0, 15, 30, 60, 120, 180, 240, 300 and 360 min after administration. Changes in IOP were given by the difference between the mean IOP value for rabbits that received each treatment and the mean IOP values from vehicle-treated rabbits at corresponding timepoints. Measurements were made without any anesthesia. The eyes were imaged at each time point to assess conjunctival hyperemia.

### 4.15. Effect of NEO on Aortic Rings Smooth Muscles

Following the intraperitoneal injection of 30 mg/kg sodium pentobarbital, the rats were dislocated and executed immediately. After opening the thoracic cavity quickly, the thoracic aorta was obtained and placed at chilled Krebs-Henseleit (K-H; pH 7.4) saturated with 95% O_2_ and 5% CO_2_. The residual blood in the K-H solution was removed, and the peripheral fat and connective tissue of the blood vessel was carefully cut off. The blood vessel was then cut into vascular rings with a length of 3–4 mm. The blood vessels were then placed in a constant temperature (37 °C) bath containing K–H solution with one end fixed to the bottom of the bath, and the other connected to a tension converter. The vessels were then continuously filled with a mixture of 95% O_2_ and 5% CO_2_. The isotonic contraction force of the blood vessels was then recorded using a PowerLab Biosignal recording system (ML0186, ADInstruments, Shanghai, China) with initial tension set to 1 g. The test was equilibrated for 15 min and repeated twice. After the maximum contraction amplitude, the medium was changed three times, the balance was adjusted to 1 g, and subsequent experiments were carried out after 30 min of stability.

For stabilization at the maximum contraction amplitude, isolated vascular rings were stimulated with 60 mM KCl before treatment with NEO, SGI-1776, and NET at final concentrations of 5, 10, 20, 40, 80 and 160 μM. A solvent was used in the same volume for the blank control group. Next, the relaxation effects of the three drugs on the hyperkalemic pre-constricted vascular ring were recorded. Additionally, to assess the effect of drug action time, 10 μM NEO, SGI-1776, and NET were added in one-time administration and recorded at 0, 5, 15, 30, 60, 90 and 120 min, respectively. Finally, the tissues of the corresponding groups were quickly frozen and stored at −80 °C and used for western blot analysis after protein extraction.

### 4.16. Statistical Analysis

The experimental data were represented as mean ± SD of at least three independent experiments. Data comparisons were undertaken using a one-way ANOVA with LSD post hoc test. *p* < 0.05 indicated statistically significant differences.

## 5. Conclusions

This study demonstrated that the natural product (NEO) targets PIM1 and inhibits ROCK2/STAT3, as well as its downstream signaling pathways, thereby suppressing TNBC cell proliferation, migration and invasion. In addition, NEO can regulate cell cycle arrest, apoptosis and autophagy. Finally, we verified that NEO suppresses IOP by relaxing the smooth muscles. These findings imply that NEO is a natural PIM1 inhibitor and is a promising therapeutic option for TNBC and glaucoma, among others.

## Figures and Tables

**Table 1:** Effects of NEO and SGI-1776 treatment on IC_50_ (μM) of cells.

Cell Name	Cell Type	NEO	SGI-1776
MDA-MB-231	Triple negative breast cancer cells	4.69 ± 0.38	4.91 ± 0.21
HEPG-2	Liver cancer cells	6.08 ± 0.64	11.92 ± 2.76
NCI-H929	Myeloma cells	7.25 ± 1.24	8.73 ± 1.46
AGS	Gastric adenocarcinoma cells	9.06 ± 0.72	7.65 ± 1.38
MCF-7	Breast cancer cells	9.51 ± 0.42	13.34 ± 1.57
H460	Large cell lung cancer cells	13.46 ± 2.04	4.93 ± 0.51
ES-2	Ovarian clear cell carcinoma cells	14.54 ± 3.65	7.36 ± 0.97
A549	Non-small cell lung cancer cells	48.28 ± 9.72	16.16 ± 7.84
MCF-10A	Normal mammary epithelial cells	6.18 ± 0.61	7.63 ± 2.04
SH-SY5Y	Neuroblastoma cells	12.61 ± 2.64	10.61 ± 0.25

## Data Availability

Data available on request.
